# Methyl 2-amino-5-chloro­benzoate

**DOI:** 10.1107/S1600536810042510

**Published:** 2010-10-31

**Authors:** Ya-Bin Shi, Song Xia, Fei-Fei He, Hai-Bo Wang

**Affiliations:** a College of Science, Nanjing University of Technology, Xinmofan Road No. 5 Nanjing, Nanjing 210009, People’s Republic of China; b College of Food Science and Light Industry, Nanjing University of Technology, Xinmofan Road No. 5 Nanjing, Nanjing 210009, People’s Republic of China

## Abstract

The title compound, C_8_H_8_ClNO_2_, is almost planar, with an r.m.s. deviation of 0.0410 Å from the plane through the non-hydrogen atoms. In the crystal structure, inter­molecular N—H⋯O hydrogen bonds link the mol­ecules into chains along the *b* axis. An intra­molecular N—H⋯O hydrogen bond results in the formation of a six-membered ring.

## Related literature

The title compound is a useful pharmaceutical inter­mediate, see: Dong & Xu (2009 ▶). For bond-length data, see: Allen *et al.* (1987 ▶).
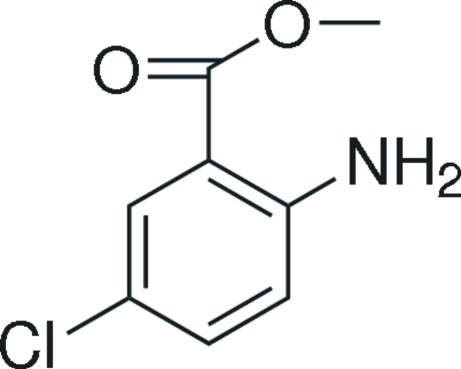


## Experimental

### 

#### Crystal data

C_8_H_8_ClNO_2_
*M* *_r_* = 185.60
Monoclinic, 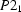

*a* = 3.9480 (8) Å
*b* = 9.0230 (18) Å
*c* = 12.018 (2) Å
β = 94.10 (3)°
*V* = 427.02 (15) Å^3^
*Z* = 2
Mo *K*α radiation
μ = 0.40 mm^−1^
*T* = 293 K
0.30 × 0.30 × 0.05 mm

#### Data collection

Enraf–Nonius CAD-4 diffractometer
Absorption correction: ψ scan (North *et al.*, 1968 ▶) *T*_min_ = 0.889, *T*_max_ = 0.980
1911 measured reflections
1663 independent reflections
1437 reflections with *I* > 2σ(*I*)
*R*_int_ = 0.035
3 standard reflections every 200 reflections  intensity decay: 1%

#### Refinement

*R*[*F* ^2^ > 2σ(*F* ^2^)] = 0.051
*wR*(*F* ^2^) = 0.160
*S* = 1.01
1663 reflections
110 parameters
1 restraint
H-atom parameters constrained
Δρ_max_ = 0.27 e Å^−3^
Δρ_min_ = −0.19 e Å^−3^
Absolute structure: Flack (1983 ▶), 777 Friedel pairs
Flack parameter: 0.30 (14)

### 

Data collection: *CAD-4 EXPRESS* (Enraf–Nonius, 1989 ▶); cell refinement: *CAD-4 EXPRESS*; data reduction: *XCAD4* (Harms & Wocadlo, 1995 ▶); program(s) used to solve structure: *SHELXS97* (Sheldrick, 2008 ▶); program(s) used to refine structure: *SHELXL97* (Sheldrick, 2008 ▶); molecular graphics: *SHELXTL* (Sheldrick, 2008 ▶); software used to prepare material for publication: *PLATON* (Spek, 2009 ▶).

## Supplementary Material

Crystal structure: contains datablocks global, I. DOI: 10.1107/S1600536810042510/bq2241sup1.cif

Structure factors: contains datablocks I. DOI: 10.1107/S1600536810042510/bq2241Isup2.hkl

Additional supplementary materials:  crystallographic information; 3D view; checkCIF report

## Figures and Tables

**Table 1:** Hydrogen-bond geometry (Å, °)

*D*—H⋯*A*	*D*—H	H⋯*A*	*D*⋯*A*	*D*—H⋯*A*
N—H0*A*⋯O2^i^	0.86	2.31	3.066 (5)	147
N—H0*B*⋯O2	0.86	2.08	2.713 (5)	129

Symmetry code: (i) 
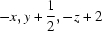
.

**Fig. 1.**
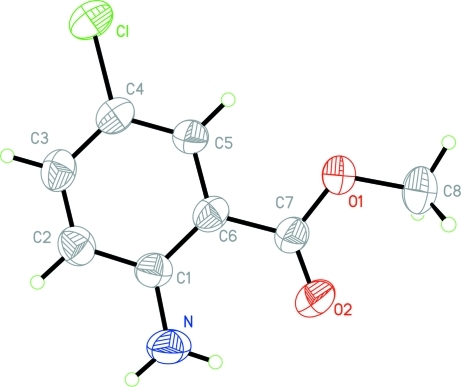
The molecular structure of the title molecule, with the atom-numbering scheme. Displacement ellipsoids are drawn at the 50% probability level.

**Fig. 2.**
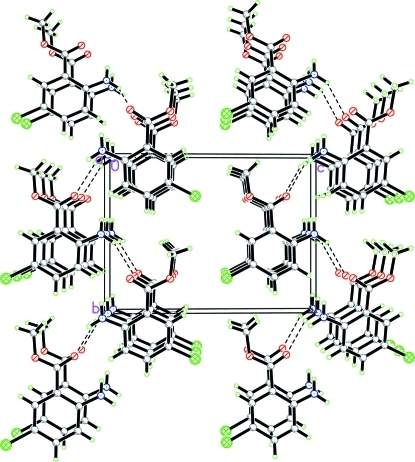
A packing diagram of (I). Hydrogen bond is shown as dashed line.

## supplementary crystallographic information

### Comment

Quinazolinones play an important role in the fields of natural products and medicinal chemistry. The title compound, methyl 2-amino-5-chlorobenzoate, (I), is a useful pharmaceutical intermediate (Dong *et al.* 2009). The molecule of (I) (Figure 1.) is almost planar (except the methyl hydrogens) with r. m. s. deviation of 0.0410 Å and the bond lengths (Allen *et al.*, 1987) and angles are within normal ranges. The intramolecular N-H···O hydrogen bond (Table 1) results in the formation of a six-membered ring (C1/C6/C7/O2/H0B/N). In the crystal structure, intermolecular N-H0A···O2 hydrogen bonds link the molecules to form a stable structure (Table 1. and Figure 2.).

### Experimental

The title compound, methyl 2-amino-5-chlorobenzoate was prepared by the literature method (Dong *et al.*, 2009). To a solution of 2-aminobenzoic acid (10 g, 66 mmol) in DMF (40 mL) was added N-halosuccinimide (66 mmol) and the reaction mixture was heated at 100 °C for 40 min, cooled to room temperature, left stand overnight, and then slowly poured into ice-water (150 mL) to precipitate a white solid. The solid was filtered, washed with water (50 mL * 3), then taken up in ethyl acetate (600 mL). The ethyl acetate solution was dried over magnesium sulfate, evaporated under reduced pressure and the residual solid was washed with ether (30 mL * 3) to afford intermediate 2-amino-5-chlorobenzoic acid. To an alcohol solution (60 mL) containing 2-amino-5-chlorobenzoic acid (20 mmol) was added thionyl chloride (60 mmol), and the resulting suspension was refluxed overnight. The solvent was evaporated followed by addition of EtOAc, washed with 10% NaOH solution, dried, filtered, and evaporated to afford the desired anthranilic acid esters methyl 2-amino-5-chlorobenzoate. Crystals suitable for X-ray analysis were obtained by slow evaporation of an methanol solution.

### Refinement

H atoms were positioned geometrically, with N-H = 0.86 Å (for NH_2_) and C-H = 0.93, 0.98 and 0.96 Å for aromatic, methine and methyl H, respectively, and constrained to ride on their parent atoms, with U_iso_(H) = xU_eq_(C,N), where x = 1.5 for methyl H and x = 1.2 for all other H atoms.

### Crystal data

**Table:**

C_8_H_8_ClNO_2_	*F*(000) = 192
*M**_r_* = 185.60	*D*_x_ = 1.444 Mg m^−^^3^
Monoclinic, *P*2_1_	Melting point: 343 K
Hall symbol: P 2yb	Mo *K*α radiation, λ = 0.71073 Å
*a* = 3.9480 (8) Å	Cell parameters from 25 reflections
*b* = 9.0230 (18) Å	θ = 10–14°
*c* = 12.018 (2) Å	µ = 0.40 mm^−^^1^
β = 94.10 (3)°	*T* = 293 K
*V* = 427.02 (15) Å^3^	Block, colourless
*Z* = 2	0.30 × 0.30 × 0.05 mm

### Data collection

**Table:**

Enraf–Nonius CAD-4 diffractometer	1437 reflections with *I* > 2σ(*I*)
Radiation source: fine-focus sealed tube	*R*_int_ = 0.035
graphite	θ_max_ = 26.0°, θ_min_ = 1.7°
ω/2θ scans	*h* = 0→4
Absorption correction: ψ scan (North *et al.*, 1968)	*k* = −11→11
*T*_min_ = 0.889, *T*_max_ = 0.980	*l* = −14→14
1911 measured reflections	3 standard reflections every 200 reflections
1663 independent reflections	intensity decay: 1%

### Refinement

**Table:**

Refinement on *F*^2^	Hydrogen site location: inferred from neighbouring sites
Least-squares matrix: full	H-atom parameters constrained
*R*[*F*^2^ > 2σ(*F*^2^)] = 0.051	*w* = 1/[σ^2^(*F*_o_^2^) + (0.1*P*)^2^ + 0.190*P*] where *P* = (*F*_o_^2^ + 2*F*_c_^2^)/3
*wR*(*F*^2^) = 0.160	(Δ/σ)_max_ < 0.001
*S* = 1.00	Δρ_max_ = 0.27 e Å^−^^3^
1663 reflections	Δρ_min_ = −0.19 e Å^−^^3^
110 parameters	Extinction correction: *SHELXL97* (Sheldrick, 2008), Fc^*^=kFc[1+0.001xFc^2^λ^3^/sin(2θ)]^-1/4^
1 restraint	Extinction coefficient: 0.103 (19)
Primary atom site location: structure-invariant direct methods	Absolute structure: Flack (1983), 777 Friedel pairs
Secondary atom site location: difference Fourier map	Flack parameter: 0.30 (14)

### Special details

**Table:**

Geometry. All esds (except the esd in the dihedral angle between two l.s. planes) are estimated using the full covariance matrix. The cell esds are taken into account individually in the estimation of esds in distances, angles and torsion angles; correlations between esds in cell parameters are only used when they are defined by crystal symmetry. An approximate (isotropic) treatment of cell esds is used for estimating esds involving l.s. planes.
Refinement. Refinement of F^2^ against ALL reflections. The weighted R-factor wR and goodness of fit S are based on F^2^, conventional R-factors R are based on F, with F set to zero for negative F^2^. The threshold expression of F^2^ > 2sigma(F^2^) is used only for calculating R-factors(gt) etc. and is not relevant to the choice of reflections for refinement. R-factors based on F^2^ are statistically about twice as large as those based on F, and R- factors based on ALL data will be even larger.

### Fractional atomic coordinates and isotropic or equivalent isotropic displacement parameters (Å^2^)

**Table:**

	*x*	*y*	*z*	*U*_iso_*/*U*_eq_	
Cl	0.5983 (3)	0.76879 (13)	0.57288 (9)	0.0669 (4)	
N	0.1559 (10)	0.5027 (4)	0.9873 (3)	0.0566 (10)	
H0A	0.1791	0.5545	1.0474	0.068*	
H0B	0.0700	0.4152	0.9888	0.068*	
O1	0.1109 (8)	0.2663 (4)	0.6830 (2)	0.0570 (7)	
C1	0.2559 (10)	0.5595 (4)	0.8894 (3)	0.0416 (8)	
O2	−0.0279 (9)	0.2648 (4)	0.8588 (2)	0.0664 (8)	
C2	0.3960 (10)	0.7029 (4)	0.8887 (4)	0.0471 (9)	
H2A	0.4190	0.7567	0.9548	0.057*	
C3	0.4982 (9)	0.7643 (5)	0.7937 (3)	0.0484 (8)	
H3A	0.5884	0.8595	0.7954	0.058*	
C4	0.4699 (10)	0.6870 (4)	0.6945 (3)	0.0459 (9)	
C5	0.3416 (10)	0.5457 (4)	0.6914 (3)	0.0437 (9)	
H5A	0.3266	0.4932	0.6246	0.052*	
C6	0.2327 (9)	0.4797 (4)	0.7884 (3)	0.0410 (8)	
C7	0.0929 (10)	0.3288 (4)	0.7827 (3)	0.0425 (8)	
C8	−0.0251 (14)	0.1186 (5)	0.6689 (4)	0.0650 (13)	
H8A	0.0020	0.0853	0.5941	0.097*	
H8B	−0.2619	0.1192	0.6822	0.097*	
H8C	0.0942	0.0528	0.7208	0.097*	

### Atomic displacement parameters (Å^2^)

**Table:**

	*U*^11^	*U*^22^	*U*^33^	*U*^12^	*U*^13^	*U*^23^
Cl	0.0859 (8)	0.0590 (6)	0.0578 (6)	−0.0183 (6)	0.0195 (5)	0.0133 (5)
N	0.082 (3)	0.049 (2)	0.0405 (18)	0.0089 (18)	0.0160 (17)	0.0031 (16)
O1	0.0802 (18)	0.0415 (13)	0.0503 (14)	−0.0169 (17)	0.0130 (13)	−0.0067 (15)
C1	0.048 (2)	0.0374 (18)	0.0394 (19)	0.0079 (16)	0.0049 (16)	0.0026 (15)
O2	0.099 (2)	0.0470 (16)	0.0564 (16)	−0.009 (2)	0.0251 (15)	0.0092 (17)
C2	0.052 (2)	0.042 (2)	0.048 (2)	0.0045 (17)	0.0046 (16)	−0.0069 (17)
C3	0.050 (2)	0.0359 (17)	0.059 (2)	−0.002 (2)	0.0056 (16)	−0.002 (2)
C4	0.048 (2)	0.043 (2)	0.048 (2)	−0.0020 (18)	0.0067 (16)	0.0090 (17)
C5	0.050 (2)	0.041 (2)	0.041 (2)	−0.0016 (16)	0.0110 (16)	−0.0016 (16)
C6	0.0412 (19)	0.0360 (18)	0.046 (2)	0.0028 (15)	0.0068 (15)	0.0057 (15)
C7	0.048 (2)	0.0347 (17)	0.045 (2)	0.0013 (16)	0.0053 (16)	0.0034 (15)
C8	0.080 (3)	0.042 (2)	0.073 (3)	−0.018 (2)	0.007 (3)	−0.008 (2)

### Geometric parameters (Å, °)

**Table:**

Cl—C4	1.744 (4)	C2—H2A	0.9300
N—C1	1.367 (5)	C3—C4	1.379 (6)
N—H0A	0.8600	C3—H3A	0.9300
N—H0B	0.8600	C4—C5	1.372 (5)
O1—C7	1.330 (5)	C5—C6	1.403 (5)
O1—C8	1.443 (5)	C5—H5A	0.9300
C1—C2	1.408 (6)	C6—C7	1.469 (5)
C1—C6	1.409 (5)	C8—H8A	0.9600
O2—C7	1.208 (5)	C8—H8B	0.9600
C2—C3	1.357 (6)	C8—H8C	0.9600
			
C1—N—H0A	120.0	C4—C5—C6	120.4 (4)
C1—N—H0B	120.0	C4—C5—H5A	119.8
H0A—N—H0B	120.0	C6—C5—H5A	119.8
C7—O1—C8	117.0 (3)	C5—C6—C1	119.6 (3)
N—C1—C2	119.1 (4)	C5—C6—C7	119.4 (4)
N—C1—C6	123.1 (4)	C1—C6—C7	121.0 (3)
C2—C1—C6	117.8 (3)	O2—C7—O1	121.9 (4)
C3—C2—C1	121.4 (4)	O2—C7—C6	125.1 (4)
C3—C2—H2A	119.3	O1—C7—C6	113.0 (3)
C1—C2—H2A	119.3	O1—C8—H8A	109.5
C2—C3—C4	120.7 (4)	O1—C8—H8B	109.5
C2—C3—H3A	119.6	H8A—C8—H8B	109.5
C4—C3—H3A	119.6	O1—C8—H8C	109.5
C5—C4—C3	120.0 (4)	H8A—C8—H8C	109.5
C5—C4—Cl	120.0 (3)	H8B—C8—H8C	109.5
C3—C4—Cl	120.0 (3)		
			
N—C1—C2—C3	−179.8 (4)	C2—C1—C6—C5	−1.3 (5)
C6—C1—C2—C3	1.6 (6)	N—C1—C6—C7	0.8 (5)
C1—C2—C3—C4	−0.5 (6)	C2—C1—C6—C7	179.3 (4)
C2—C3—C4—C5	−0.9 (6)	C8—O1—C7—O2	0.9 (6)
C2—C3—C4—Cl	179.3 (3)	C8—O1—C7—C6	−179.1 (4)
C3—C4—C5—C6	1.2 (6)	C5—C6—C7—O2	−175.1 (4)
Cl—C4—C5—C6	−179.0 (3)	C1—C6—C7—O2	4.3 (6)
C4—C5—C6—C1	0.0 (6)	C5—C6—C7—O1	4.9 (5)
C4—C5—C6—C7	179.3 (3)	C1—C6—C7—O1	−175.7 (3)
N—C1—C6—C5	−179.8 (4)		

### Hydrogen-bond geometry (Å, °)

**Table:**

*D*—H···*A*	*D*—H	H···*A*	*D*···*A*	*D*—H···*A*
N—H0A···O2^i^	0.86	2.31	3.066 (5)	147
N—H0B···O2	0.86	2.08	2.713 (5)	129

Symmetry codes: (i) −*x*, *y*+1/2, −*z*+2.

## References

[bb1] Allen, F. H., Kennard, O., Watson, D. G., Brammer, L., Orpen, A. G. & Taylor, R. (1987). *J. Chem. Soc. Perkin Trans. 2*, pp. S1–19.

[bb2] Dong, W. L. & Xu, J. Y. (2009). *Chin. J. Chem.* **27**, 579–586.

[bb3] Enraf–Nonius (1989). *CAD-4 EXPRESS* Enraf–Nonius, Delft. The Netherlands.

[bb4] Flack, H. D. (1983). *Acta Cryst.* A**39**, 876–881.

[bb5] Harms, K. & Wocadlo, S. (1995). *XCAD4* University of Marburg, Germany.

[bb6] North, A. C. T., Phillips, D. C. & Mathews, F. S. (1968). *Acta Cryst.* A**24**, 351–359.

[bb7] Sheldrick, G. M. (2008). *Acta Cryst.* A**64**, 112–122. 10.1107/S0108767307043930 18156677

[bb8] Spek, A. L. (2009). *Acta Cryst.* D**65**, 148–155. 10.1107/S090744490804362X PMC2631630 19171970

